# CAR-T治疗复发/难治急性B淋巴细胞白血病的长期生存及影响因素的临床分析

**DOI:** 10.3760/cma.j.issn.0253-2727.2023.10.002

**Published:** 2023-10

**Authors:** 一 王, 秋英 高, 晖 王, 玎 张, 瑛 高, 玉迪 苗, 欣辉 翟, 星星 胡, 杏丽 茹, 维华 张

**Affiliations:** 陕西省人民医院血液内科，西安 710068 Department of Hematology, Shaanxi Provincial People's Hospital, Xi'an 710068, China

**Keywords:** 急性B淋巴细胞白血病, 嵌合抗原受体T细胞, 复发/难治, 预后, Acute lymphoblastic leukemia, Chimeric antigen receptor-modified T cell, Relapsed/refractory, Prognosis

## Abstract

**目的:**

分析嵌合抗原受体T细胞（CAR-T）治疗复发/难治急性B淋巴细胞白血病（R/R B-ALL）获完全缓解（CR）患者的长期生存及其影响因素。

**方法:**

收集2015年5月至2018年7月就诊于陕西省人民医院接受靶向CD19的CAR-T细胞治疗并获得CR的R/R B-ALL患者的临床信息。采用Kaplan-Meier法评估患者的总生存（OS）和无白血病生存（LFS），并通过Cox等比例风险回归分析CAR-T治疗后患者预后的影响因素。

**结果:**

纳入的38例R/R B-ALL患者中，男性患者21例，中位年龄25（6～59）岁，中位OS时间为18（95％*CI* 3～33）个月。多因素Cox回归分析显示：MLL-AF4融合基因阳性是OS及LFS的独立危险因素（OS：*HR*＝4.888，95％ *CI* 1.375～17.374，*P*＝0.014；LFS：*HR*＝6.683，95％*CI* 1.815～24.608，*P*＝0.004）；接受维持治疗是OS及LFS的独立保护因素（OS：*HR*＝0.153，95％*CI* 0.054～0.432，*P*<0.001；LFS：*HR*＝0.138，95％*CI* 0.050～0.382，*P*<0.001）；MRD转阴患者LFS获益（*HR*＝0.209，95％*CI* 0.055～0.797，*P*＝0.022），但OS差异无统计学意义（*P*＝0.111）；具有高肿瘤负荷的患者在0.1的水平上是OS及LFS的危险因素（OS：*HR*＝2.662，95％*CI* 0.987～7.184，*P*＝0.053；LFS：*HR*＝2.452，95％*CI* 0.949～6.339，*P*＝0.064）。

**结论:**

高肿瘤负荷和高危遗传学可能会影响R/R B-ALL患者接受CAR-T治疗后的长期生存，予以来那度胺为基础的维持治疗有可能改善患者的长期预后。

## Abstract (en)

**Objective:**

To analyze the survival and influencing factors of chimeric antigen receptor（CAR）T-cell therapy in relapsed/refractory acute B-cell lymphoblastic leukemia（R/R B-ALL）.

**Methods:**

Clinical information of patients who received CAR-T-cell therapy and achieved complete remission of R/R B-ALL between May 2015 and June 2018 at the Shaanxi Provincial People's Hospital was obtained. Kaplan-Meier analysis was used to evaluate the overall survival（OS）and leukemia-free survival（LFS）times of patients, and Cox regression analysis was performed to analyze the prognostic factors that affect patient survival after CAR-T therapy.

**Results:**

Among the 38 patients with R/R B-ALL, 21 were men, with a median age of 25（6–59）years and a median OS time of 18（95％ *CI* 3–33）months. Multivariate Cox regression analysis showed that positive MLL-AF4 fusion gene expression was an independent risk factor for OS and LFS（OS: *HR*＝4.888, 95％ *CI* 1.375–17.374, *P*＝0.014; LFS: *HR*＝6.683, 95％ *CI* 1.815–24.608, *P*＝0.004）. Maintenance therapy was a protective factor for OS and LFS（OS: *HR*＝0.153, 95％*CI* 0.054–0.432, *P*<0.001; LFS: *HR*＝0.138, 95％*CI* 0.050–0.382, *P*<0.001）. In patients with MRD negative conversion, LFS benefit（*HR*＝0.209, 95％ *CI* 0.055–0.797, *P*＝0.022）and OS difference was statistically insignificant（*P*＝0.111）. Moreover, patients with high tumor burden were risk factors for OS and LFS at the level of 0.1（OS: *HR*＝2.662, 95％ *CI* 0.987–7.184, *P*＝0.053; LFS: *HR*＝2.452, 95％ *CI* 0.949–6.339, *P*＝0.064）.

**Conclusion:**

High tumor burden and high-risk genetics may affect the long-term survival rate of patients with R/R B-ALL receiving CAR-T, and lenalidomide-based maintenance therapy may improve their prognosis.

急性B淋巴细胞白血病（B-ALL）是一种起源于B细胞系的恶性血液病，其在细胞分化发育过程中多表达膜抗原CD19，多发于儿童、青少年及老年人。多数儿童和青少年B-ALL经过规范的诊治可以治愈，但是复发/难治B-ALL（R/R B-ALL）预后则不佳。嵌合抗原受体T细胞（CAR-T细胞）治疗是一种新型的细胞免疫治疗方法，现有研究显示CD19 CAR-T对R/R B-ALL具有较好的临床疗效，客观缓解率可达到70％～90％，但是多数患者在CAR-T治疗后1年内复发[1]。因此CAR-T治疗缓解后的治疗和管理逐渐被重视，影响CAR-T治疗后生存的因素也被持续关注。本研究我们分析接受CAR-T治疗后获得完全缓解（CR）的38例R/R B-ALL患者长期生存情况及其影响因素，报道如下。

## 病例与方法

一、病例

本研究为回顾性队列研究，纳入2015年5月至2018年7月陕西省人民医院血液内科接受靶向CD19 CAR-T治疗后获得CR的R/R B-ALL患者。R/R B-ALL的诊断符合《中国成人急性淋巴细胞白血病诊断与治疗指南（2021年版）》[2]及NCCN儿童B-ALL的标准，所有患者骨髓流式细胞免疫分型白血病优势表达CD19。研究经陕西省人民医院医学伦理委员会批准（批件号：SPPH-LLBG-17-3.2）。

二、CAR-T细胞制备

通过细胞单采机收集自体或者来自供者的外周血单个核细胞，利用磁珠分选进行CD3^+^细胞的分选后，体外转导慢病毒介导的CAR-T结构，共刺激分子为41-BB，体外培养扩增，检测CAR-T转导率、CD4和CD8比例，有无白血病细胞污染，并进行内毒素、细菌及支原体检测。

三、CAR-T细胞输注

所有患者均进行清除淋巴细胞的预处理方案，包括环磷酰胺（CTX）单药方案（25例）：CTX 500 mg·m^−2^·d^−1^，−5～−3 d；氟达拉滨（Flu）+环磷酰胺预处理方案（FC方案）（13例）：Flu 25 mg·m^−2^·d^−1^，−5～−3 d，CTX 300 mg·m^−2^·d^−1^，−5～−3 d；预处理后2～5天后分次或者一次性输入CAR-T细胞。5例（13.2％）患者CAR-T细胞1次输注，18例（47.4％）患者细胞分别以40％和60％的细胞数分2次输注，15例患者（39.5％）分别以20％、30％和50％的CAR-T细胞数分3次输注。CAR-T细胞中位输注量为 6.4（4.2～17.7）×10^6^/kg。

四、不良反应的评估及处理

细胞因子释放综合征（CRS）和免疫效应细胞相关神经毒性综合征（ICANS）按照Lee等研究和NCCN指南[4]–[5]来进行评估与分级。输注后分别监测不同时间节点的生命体征、意识状态、肝功能、肾功能、出凝血指标、血清炎症因子水平、血常规、心电图、心肌酶谱以及N端脑钠肽水平等。

所有患者输注CAR-T细胞后的处理以对症支持治疗为基础。体温持续高于38.5 °C，予以非甾体类抗炎药退热处理；低氧血症予以吸氧支持，仅有低血压予以补液或者联合升压药物治疗，出现明显水肿则予以利尿治疗，心率明显增加予以降心率治疗；丙戊酸钠预防癫痫发生；常规治疗不能缓解的低血压、低氧血症及持续高热或者意识障碍者予地塞米松每次5～10 mg，每日1～2次，连续3～5 d治疗。

五、后续治疗

18例（47.3％）患者在CAR-T治疗后3个月开始接受维持治疗，根据患者Ph染色体是否阳性选择维持治疗方案，其中10例（55.6％）Ph^−^患者接受来那度胺联合6-巯基嘌呤（6-MP）治疗，8例（44.4％）Ph^+^患者接受来那度胺联合达沙替尼治疗。4例（10.5％）患者在CAR-T治疗6个月后接受异基因造血干细胞移植。

六、疗效评估及随访

疗效评估：按照文献[2]–[3]标准进行临床疗效评估，Ph^+^或MLL-AF4融合基因阳性的患者以实时荧光定量PCR（qPCR）定量检测和多色流式细胞术检测，其他患者均用流式细胞术检测微小残留病（MRD），疗效判断MRD阴性的CR，MRD阳性的CR。

随访截止时间为2023年7月20日，期间死亡患者记录死亡日期。通过门诊、电话、短信及微信等方式进行随访。输注后2年内每3个月随访，2～5年每半年随访一次，5年后每年随访一次。随访时进行骨髓涂片、流式检测；髓外浸润的判断：中枢浸润、睾丸浸润以及肝脏等部位的浸润，结合骨髓中白血病比例、脑脊液、组织活检、PET/CT、全身CT等检查确定。总生存（OS）时间定义为开始CAR-T细胞输注至患者死亡或随访终点的时间。无白血病生存（LFS）时间定义为从CAR-T输注后达到CR至白血病复发、死亡或随访终点的时间。

七、统计学处理

所有统计分析均使用SPSS 20.0和风锐统计软件。采用Kaplan-Meier法绘制生存曲线，单因素及多因素分析采用Cox等比例风险回归模型，单因素分析中*P*<0.05的变量纳入多因素分析。以双侧*P*<0.05为差异有统计学意义。

## 结果

一、患者基本临床特征

共有38例R/R B-ALL患者CD19 CAR-T细胞治疗后2周或4周疗效评估，其中男21例，女17例，中位年龄25（6～59）岁。难治20例（52.6％），复发18例（47.4％）。13例（34.2％）患者合并髓外病变，13例（34.2％）Ph染色体阳性；输注前骨髓白血病细胞比例中位数为25％（1.5％～97％），11患者例骨髓白血病细胞比例≥30％，提示为高肿瘤负荷；所有患者均进行基因突变检测，其中4例（10.5％）合并T315I突变，5例（13.2％）MLL-AF4融合基因阳性；既往5例患者（13.2％）获得CR后接受异基因造血干细胞移植，无患者既往接受过CAR-T治疗。所有患者均经过标准VDLP或VDCP方案联合治疗，后续均进行大剂量MTX、Hyper-CVAD等常规方案治疗；合并Ph^+^患者CAR-T治疗前均接受过酪氨酸激酶抑制剂的联合治疗。

二、不良反应

33例（86.4％）出现1～2级CRS反应，5例（13.2％）出现3级CRS反应；3例（7.9％）出现ICANS，其中2例（5.2％）1级，1例（2.6％）2级。出现1～2级CRS仅予以对症治疗，3级CRS和任意级别的ICANS反应后予以对症支持联合皮质激素治疗，1个月内所有患者CRS和ICANS反应均得到控制。

三、生存分析

中位随访69（95％*CI* 62～76）个月，38例CD19 CAR-T治疗后获得CR的R/R B-ALL患者，24例死亡，中位OS时间为18（95％*CI* 3～33）个月，1、3、5年OS率分别为（65.8±7.7）％、（47.4±8.1）％、（36.8±7.8）％；中位LFS时间为16（95％*CI* 0～37）个月，1、3、5年LFS率分别为（55.3±8.1）％、（47.4±8.1）％、（36.8±7.8）％（图1）。13例（34.2％）患者在CAR-T输注5年后持续缓解，按照白血病的疗效判断已经达到临床治愈。

**图1:**
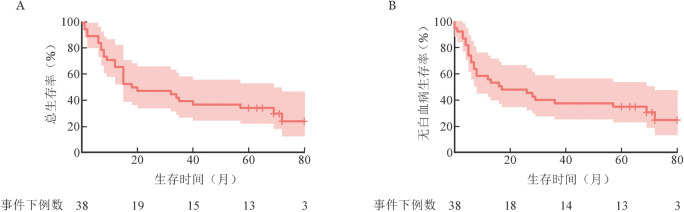
38例CAR-T治疗后获完全缓解的复发/难治急性B淋巴细胞白血病患者总生存（A）与无白血病生存（B）曲线

四、预后影响因素分析

单因素分析显示，高肿瘤负荷、MLL-AF4融合基因阳性是OS和LFS的危险因素，而合并髓外病变、CAR-T治疗后接受维持治疗及CAR-T治疗后1个月MRD转阴是OS和LFS的保护因素；年龄、性别、Ph染色体阳性与否、预处理方案、是否多次输注以及CAR-T治疗后是否接受造血干细胞移植对患者的生存无明显影响（*P*>0.05）。（表1）。

**表1:** 影响CAR-T治疗后获完全缓解的R/R B-ALL患者预后的单因素及多因素分析

因素	单因素分析				多因素分析			
	总生存		无白血病生存		总生存		无白血病生存	
	*HR*（95% *CI*）	*P*值	*HR*（95% *CI*）	*P*值	*HR*（95% *CI*）	*P*值	*HR*（95% *CI*）	*P*值
年龄（≤30岁/>30岁）	0.856（0.373~1.964）	0.714	0.874（0.381~2.006）	0.751				
性别（男性/女性）	0.987（0.438~2.225）	0.975	1.007（0.446~2.270）	0.987				
Ph染色体（阴性/阳性）	1.264（0.566~2.823）	0.568	1.180（0.528~2.637）	0.687				
高肿瘤负荷（否/是）	2.912（1.263~6.715）	0.012	2.881（1.255~6.617）	0.013	2.662（0.987~7.184）	0.053	2.452（0.949~6.339）	0.064
髓外病变（否/是）	0.364（0.144~0.920）	0.033	0.386（0.153~0.975）	0.044	0.671（0.236~1.907）	0.454	0.795（0.284~2.225）	0.662
MLL-AF4融合基因（阴性/阳性）	4.824（1.740~13.375）	0.002	4.961（1.771~13.896）	0.002	4.888（1.375~17.374）	0.014	6.683（1.815~24.608）	0.004
预处理方案（CTX/FC）	0.718（0.297~1.738）	0.463	0.618（0.256~1.492）	0.284				
多次输注（否/是）	0.520（0.231~1.169）	0.114	0.591（0.264~1.324）	0.201				
维持治疗（否/是）	0.164（0.065~0.414）	<0.001	0.149（0.059~0.377）	<0.001	0.153（0.054~0.432）	<0.001	0.138（0.050~0.382）	<0.001
MRD转阴（否/是）	0.173（0.060~0.496）	0.001	0.130（0.040~0.418）	0.001	0.379（0.115~1.248）	0.111	0.209（0.055~0.797）	0.022
桥接HSCT（否/是）	0.717（0.213~2.410）	0.590	0.815（0.243~2.735）	0.741				

注 CAR-T：嵌合抗原受体T细胞；R/R B-ALL：复发/难治急性B淋巴细胞白血病；MRD：微量残留病；HSCT：造血干细胞移植

将单因素分析有统计学意义的变量纳入多因素Cox回归模型，结果见表1，MLL-AF4融合基因阳性是OS（*HR*＝4.888，95％ *CI* 1.375～17.374，*P*＝0.014）及LFS（*HR*＝6.683，95％*CI* 1.815～24.608，*P*＝0.004）的独立危险因素；接受维持治疗是OS（*HR*＝0.153，95％*CI* 0.054～0.432，*P*<0.001）及LFS（*HR*＝0.138，95％*CI* 0.050～0.382，*P*<0.001）的保护因素；MRD转阴患者LFS获益（*HR*＝0.209，95％*CI* 0.055～0.797，*P*＝0.022）但OS差异无统计学意义（*P*＝0.111）。具有高肿瘤负荷的患者在0.1的水平上是OS（*HR*＝2.662，95％*CI* 0.987～7.184，*P*＝0.053）及LFS（*HR*＝2.452，95％*CI* 0.949～6.339，*P*＝0.064）的危险因素。

## 讨论

本研究我们回顾性分析了2015—2018年期间在我中心接受CAR-T治疗后达到完全缓解的38例R/R B-ALL患者的临床资料，并分析了影响OS和LFS的因素。多数R/R B-ALL患者接受靶向CD19 CAR-T治疗后可获得MRD转阴的CR，但是CAR-T治疗后多数患者病情也会复发进展[6]–[7]，因此临床研究结果显示CAR-T后序贯造血干细胞移植可获得较好的临床疗效[8]–[9]。但是仔细分析这些研究结果发现，CAR-T治疗后进行造血干细胞移植患者观察时间较短而且多是低肿瘤负荷的患者生存获益。一项超过5年的临床观察结果显示异基因造血干细胞移植并没有提高接受CAR-T治疗的R/R B-ALL患者的LFS[10]。因此，R/R B-ALL 患者接受CAR-T治疗后进行造血干细胞移植的必要性有待进一步商榷。

血脑屏障的存在导致普通的化疗药物和单抗等药物不能进入中枢，合并中枢浸润的ALL大多预后不良，大剂量甲氨蝶呤、全脊髓放化疗和异基因造血干细胞移植等方法的临床治疗效果亦不佳[2]。此外，合并睾丸和其他软组织浸润的B-ALL预后欠佳。CAR-T细胞可穿透睾丸和血脑屏障，并能在这些部位发挥有效的抗白血病作用[11]–[12]，临床研究结果显示CAR-T治疗合并中枢浸润的B细胞肿瘤具有较好的临床效果[13]–[14]。在单因素分析中，我们发现合并髓外病变的R/R B-ALL患者接受CAR-T治疗后的长期预后明显优于无髓外病变的患者。目前对合并髓外病变的更益于CAR-T治疗原因的相关文献不多，可能的原因是这类患者的白血病细胞增殖较慢，使CAR-T细胞在这些组织内有充足的时间增殖，持续杀伤白血病细胞，最后彻底清除体内残存的白血病细胞，使患者获得长期缓解。

本研究中有18例（47.4％）患者经过CAR-T治疗3～6个月后采取了以来那度胺为基础的维持治疗，来那度胺既是抗肿瘤药物，也是免疫调节剂，一些临床前研究发现，来那度胺可提高CAR-T增殖，减少CAR-T细胞的耗竭[15]–[16]，也有少量的临床结果提示B细胞淋巴瘤经过CAR-T治疗后，持续予以小剂量的来那度胺可有效延长患者的生存期[17]。本研究发现接受CAR-T治疗后继续来那度胺为基础的维持治疗的患者，可以获得更好的长期生存。尽管不少研究表明，CAR-T治疗更有利于儿童和青少年患者，联合氟达拉滨处理对患者的生存获益，而合并Ph^+^的患者进行CAR-T治疗则预后较差[6],[18]–[19]。但本研究发现年龄、是否联合氟达拉滨的预处理以及是否合并Ph^+^不影响患者的长期生存，可能的原因是患者接受了以来那度胺为基础的维持治疗。相比造血干细胞移植和联合化疗相比，以来那度胺为基础的维持治疗简单易行。但是本研究样本量尚小，需要更多研究证实来那度胺是否可以有效改善CAR-T治疗后患者的长期生存。

本研究的结果提示接受CAR-T治疗的R/R B-ALL患者，如果合并MLL-AF4融合基因、高肿瘤负荷以及CAR-T后MRD阳性等因素可能更容易复发。随着新药和抗CD19/CD22单抗的不断应用于临床，对于高肿瘤负荷及合并MLL-AF4基因的患者，可以在CAR-T治疗前桥接新药或者单抗的方法减低白血病细胞比例，尽量使CAR-T后达到MRD转阴，再予来那度胺维持治疗，这样可能会获得更好的临床疗效。

综上，本研究结果初步显示高肿瘤负荷、MLL-AF4融合基因阳性可能是影响R/R B-ALL患者生存的危险因素，来那度胺为基础的维持治疗是生存的保护因素。但是本研究是一项单中心回顾性研究，样本量有限。因而，需要多中心、样本量更大的前瞻性临床研究来进一步探究和验证上述结论。
